# Formation of heterotic pools and understanding relationship between molecular divergence and heterosis in pearl millet [*Pennisetum glaucum* (L.) R. Br.]

**DOI:** 10.1371/journal.pone.0207463

**Published:** 2019-05-07

**Authors:** Satbeer Singh, Shashi Kumar Gupta

**Affiliations:** 1 International Crops Research Institute for the Semi-Arid Tropics, Patancheru, Telangana, India; 2 Department of Genetics and Plant Breeding, Chaudhary Charan Singh Haryana Agricultural University, Hisar, India; HudsonAlpha Institute for Biotechnology, UNITED STATES

## Abstract

The present investigation was made to generate information on the heterotic pools amongst pearl millet hybrid parents. A set of 17 representative parents was selected from a diverse set of 147 hybrid parents using SSR based genetic distance (GD) and clustering pattern; 136 hybrids were developed in diallel fashion and evaluated at two locations in India. Moderate positive significant correlation (r = 0.37, p<0.01) and (r = 0.33, p<0.01) was found between GD and mid-parent heterosis (MPH) and better-parent heterosis (BPH), respectively, for grain yield for all the hybrids. Higher correlation between genetically closer individuals was observed for grain yield heterosis when the parents of B- and R- crosses had lesser genetic distance (<0.68 GD) in comparison to those parental combinations having GD higher than 0.68, indicating that the GD based predictions for grain yield are better when the parents are genetically related than when they are genetically diverse. In this study, all the pearl millet hybrid parents seems to exist in two broad-based heterotic pools; one each represented by seed and restorer parents as B × R hybrids showed highest mean heterosis for grain yield than either of B × B or R × R crosses. Further, four heterotic pools have been identified in this diverse set of hybrid parents of pearl millet, two each for seed parents (HPB1 and HPB2) and for restorer parents (HPR3 and HPR4). Among these, HPB1 × HPR3 was identified having the highest heterotic level, and could be further used to develop higher yielding pearl millet hybrids.

## Introduction

Pearl millet [*Pennisetum glaucum* (L.) R. Br] has ability to grow in the environments of low and erratic rainfall, high temperatures and low soil fertility and is the main source of food and fodder for the poor farming communities in the semi-arid tropics of Asia and Africa. It is cultivated on about 30 m ha globally, of which about 9 m ha is in India, with about 70% of pearl millet area grown under hybrids (about 6 m ha) [1]. Successful deployment of hybrids in India led to the phenomenal increase in the productivity of pearl millet from 305 kg ha^-1^ in 1950s to the present yields of 1132 kg ha^-1^ [2]. Pearl millet breeding program at International Crops Research Institute for the Semi-Arid Tropics (ICRISAT) India, and other programs in the public and private sector in India are continuously engaged in enhancing genetic diversity of hybrid parents utilizing a significant amount of breeding material of African and Asian origin. To continue the momentum of genetic gains in this crop, information on the heterotic pool patterns in existing germplasm/ pool of hybrid parents and on the heterosis prediction are needed. Several concepts have been proposed to increase heterosis further; one of them is to increase heterozygosity by crossing genetically distinct parental materials, *i*.*e*. materials belonging to the distinct heterotic pools [3]. To achieve this, available germplasm needs to be organized into heterotic pools to increase the efficiency of any hybrid breeding program [4]. Importance of formation of heterotic pools has been suggested in several of the earlier conducted studies in many different crops, like in maize (*Zea mays*) [5–8], *triticale (Triticosecale)* [9], sunflower (*Helianthus annuus*) [10], sorghum (*Sorghum bicolor*) [11] and in rice (*Oryza sativa*) [12–13]. In pearl millet, a recent study investigated combining ability patterns in West African population hybrids but couldn’t come out with clear heterotic groups [14], while the other based on SSR’s suggested existence of heterotic pools [15].

Heterotic pools can be identified by evaluating testcross performance among available germplasm in the field trials. However, the large number of hybrid parents available in any breeding program makes the evaluation of all the possible crosses impractical. Hence, to overcome this problem, Melchinger [16] suggested (i) clustering germplasm based on genetic similarities using molecular markers, (ii) selecting representative genotypes from each subgroup, (iii) evaluating crosses among the representative genotypes in field trials, and (iv) finally identify heterotic pools based on the *per se* performance, combining ability and heterosis.

Hence, clustering of available germplasm into groups is a prerequisite to formulate working heterotic pools in any crop, which can be made possible through precise phenotyping and/or genotyping of germplasm. In pearl millet, Stich *et al*., [17] was of the view that it might be difficult to classify germplasm into groups based only on the phenotypic evaluation of testcrosses due to the involvement of a large number of germplasm lines in this crop. Alternatively, some of the molecular marker based investigations on genetic diversity in pearl millet were able to well-characterize the germplasm into genetically similar groups [15, 17–21].

In addition to the approach of formation of heterotic pools to increase the efficiency of the breeding program, the other strategy could be through enhancement of breeders’ ability to predict heterosis. It could enable the breeder to evaluate larger number of hybrid parents, shorten the testing structure of the breeding program through initial accurate selection of optimal combinations and possibly reduce the cost of trial evaluation and combination testing. The use of molecular markers for prediction of heterosis on the basis of genetic diversity of parental lines was earlier suggested in several other crops, like in rice [12–13,22]; wheat (*Triticum aestivum*) [23]; rapeseed (*Brassica napus*) [24]; soybean (*Glycine max*) [25]; sunflower [26] and maize [27–28]. However, the results of several other studies were contradictory with respect to the relationship between genetic distances and heterosis [29–34].

Hence, to aid the breeders to enhance genetic gain in pearl millet, the present investigation was conducted i) to identify the heterotic pools in hybrid parents of pearl millet and ii) to determine the association between the molecular divergence and heterosis/hybrid performance.

## Materials and methods

### Plant material and SSR genotyping

A set of 150 pearl millet hybrid parents involving 75 seed parents (B-lines) and 75 restorer parents (R-lines) was used in the present study. This included 120 hybrid parents (60 B- and 60 R-lines) from ICRISAT, Patancheru and 30 (15 B- and 15 R-lines) from CCS HAU, Hisar (S1 Table). This set of parents was selected based on the diverse parentage in pedigrees considering that it well represents available diversity in these programs. B-lines were coded from B-01 to B-75 and R-lines from R-01 to R-75. Tift 23D_2_B_1_, a maintainer of A_1_ CMS system was used as a reference genotype, which was bred by introducing d_2_ dwarfing gene in the genetic background of Tift 23B_1_ at Tifton, GA, USA. DNA of each parent along with Tift 23D_2_B_1_ was isolated from approximately 100 mg of the fresh leaf material of 20 days old seedlings using the NucleoSpin 96 Plant II kit. Fifty-six SSR markers (S2 Table), identified as highly polymorphic and found distributed over all the seven linkage groups based on earlier studies [35–38] were used. The polymerase chain reactions (PCR) were performed on a GeneAmp 9700 thermal cycler (Applied Biosystems) in 10 μl reaction mixtures containing 10 ng of template DNA, 10x Kapa*Taq*Polymerase buffer with MgCl_2_, 1 mM of each deoxyribonucleotide triphosphates (dNTPs) and 0.25 U of *Taq*DNA polymerase in 384-well PCR plates. Two pM μl^-1^ of forward and four pM μl^-1^ of reverse primers were added to each PCR reaction mixture. Polymerase chain reaction program cycle parameters included an initial denaturation step of 5 min at 94°C, followed by ten cycles of 25s at 94°C of denaturation, 20s at 64°C of primer annealing which was decreased by 1°C with each cycle upto 54°C and 30s at 72°C of extension step. This was followed by 37 cycles of 25s at 94°C, 20s at 56°C and 30s at 72°C, with a final extension step of 72°C for 20 min.

After confirmation of amplification on 10–15 random samples per loci on agarose gel, PCR product of three to four different loci were pooled based on the size of amplicon and on the usage of fluorophore dye. One microliter (μL) of the resulting mixture of the PCR product pool was combined with 7 μL of HiDi loading buffer (containing formamide), 0.1 μL of the LIZ-labeled [500 (-250)] internal size standard and 3.9 μL of distilled water. Samples were denatured for 5 min at 95°C, quickly cooled on ice and size separated based on capillary electrophoresis using Applied Biosystems 3700 automatic DNA analyzer. Fragments size in base pairs were scored manually based on the SSR repeat motif and the relative migration of internal size standard using the software Genemapper 4.0 (Applied Biosystems).

### Parent selection and hybrid development

**Fig 1:**
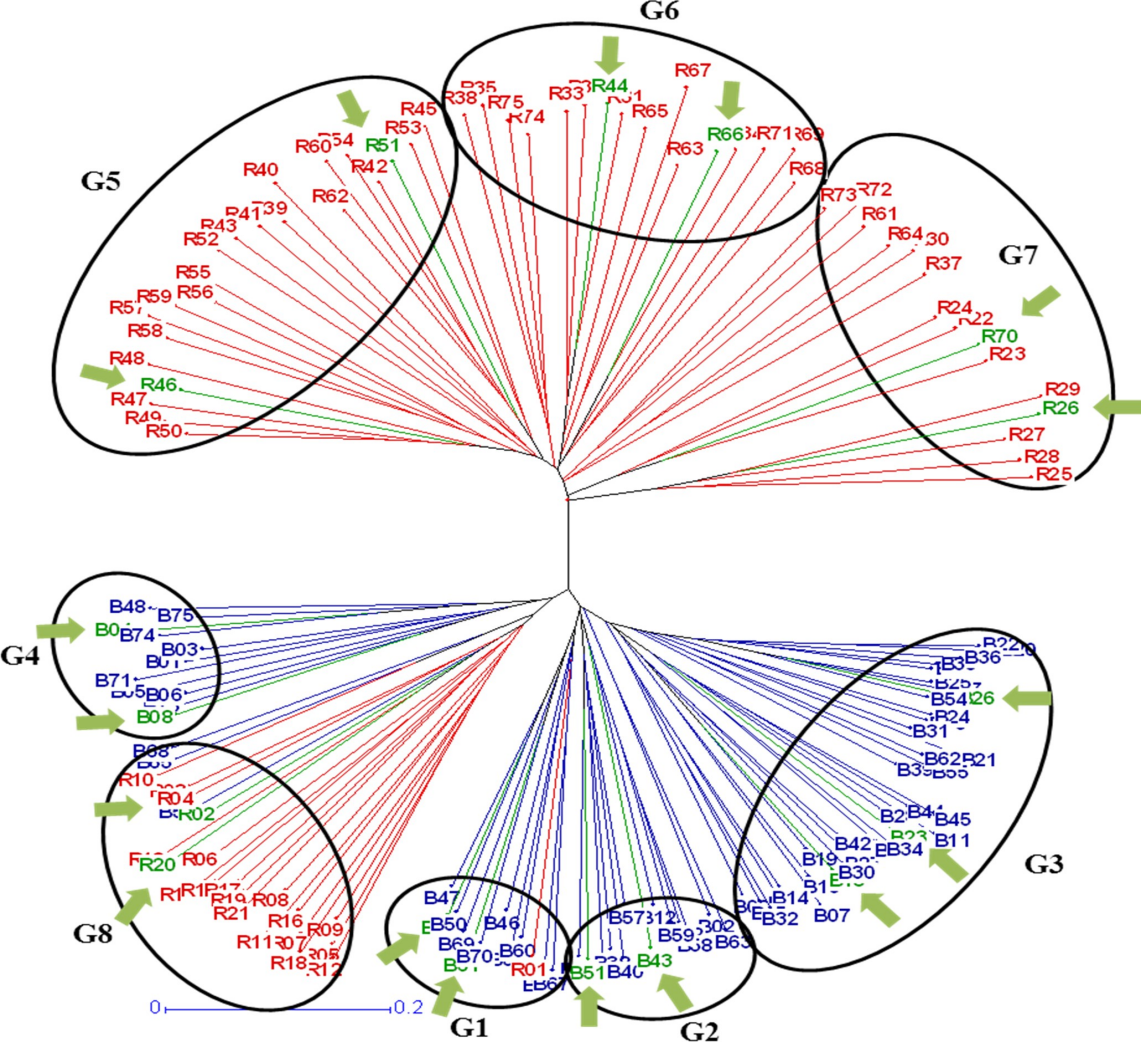
Clustering of 147 hybrid parents into 8 groups using SSRs, and selection of representative 17 parents from different marker based groups (as shown by arrow mark).

Genetic distance (GD) was estimated based on the simple matching allele frequency and the cluster diagram was developed for all the 147 hybrid parents using DARwin-5.0 software [39]. The grouping of B- and R-lines into clusters was done at 5% dissimilarity level. Clustering pattern delineated all the 147 parents into eight groups (designated as G1 to G8) (Fig 1), while two B-lines and one R-line couldn’t be analyzed due to more than 20% missing data. Representative lines for each of these eight groups were identified on the basis of total number of parents falling in that respective group and also considering genetic distance (GD) of the parent in the respective group. Based on the number of lines a group had, the groups were characterized as: large (>20% of the total lines; more than 30 lines), and small (<20% lines; less than 30 lines). As a rule, 3 and 2 representaive lines were identified from these large and small groups, respectively (S3 Table). In case when two lines were to be selected from a group, one having higher and the other having lower GD with respect to all other lines than the average GD of the specific group were selected to ensure that both of them are diverse and represent the group at the same time. In case of three lines, one line having GD equivalent to the the average GD with respect to all other lines of the specific group was selected, and rest of the two were selected following method as mentioned earlier in case of two lines. Thus, 17 pearl millet hybrid parents involving 9 seed parents (B-lines) and 8 restorer parents (R-lines) were selected to represent all the hybrid parents. Genetic distance (GD) was estimated based on the simple matching allele frequency and cluster diagram was developed for the selected set of 17 parents in DARwin-5.0 software [39]. Hybrids were developed using these 17 parents following half diallel mating design [40]. A total of 136 hybrids (36 B × B, 28 R × R and 72 B × R) were developed during the summer season of 2015 at ICRISAT, Patancheru. Pearl millet being a highly cross-pollinated crop due to its protogynous habit, so all the seed generated by crossing any two parents (B x R or B x B or R x R) was expected to be true hybrid seed. Seventy two B x R hybrids were investigated for the formation of heterotic pools, while 36 B x B and 28 R x R hybrids were developed to provide information on the line breeding strategy for improvement of hybrid parents.

### Experiment layout

A trial comprising of 136 hybrids, 17 parents and four standard hybrid checks (HHB 67 Improved, HHB 226, Pioneer 86M88 and Pro Agro 9444) was evaluated during rainy season (July to October) of 2015 at two locations, CCS HAU-Hisar (29^0^15’ N latitude, 75^0^7’ E longitude and 215.2 m altitude) and ICRISAT-Patancheru (17°53’ N latitude, 78°27’ E longitude and 545 m altitude) in alpha lattice design with two replications. These two locations in this study represented two different zones (zoning pattern as used by AICPMIP-All India Coordinated Pearl Millet Improvement Project) of pearl millet cultivation in India i) A-zone (Hisar) and ii) B-zone (Patancheru) based on the rainfall pattern and local adaptation of crop, which represents two clear cut separate mega-environments [41]. The hybrids and parents were evaluated in separate blocks planted side by side to avoid the suppressive effect of hybrids on parents due to their taller height. At Patancheru, each entry was planted in two rows of 4m length, rows spaced 75 cm apart and plants were maintained 15 cm apart. At Hisar, each entry was planted in four rows of 4m length, rows spaced 50 cm apart and plants were maintained 12 cm apart. All the recommended agronomic practices were followed at both the locations for good crop growth. All the panicles in a plot were harvested for each entry separately. The harvested material was sundried for 10 to 15 days, threshed and recorded for grain yield in kilogram per plot and converted to grain yield in kilogram per hectare.

### Statistical analysis

#### Phenotypic analysis

Variance components were estimated following linear mixed model for multi environment diallel method-II (half diallel with parents) [40] in SAS v9.3 [42]. Heterosis for grain yield was estimated as i) mid-parent heterosis (MPH) = 100 × (F_1_ –MP)/MP; ii) better-parent heterosis (BPH) = 100 × (F_1_ –BP)/BP; and iii) standard heterosis (SH) = 100 × (F_1_ –SC)/SC; where F_1_ is the hybrid yield, MP is the mean yield of both the parents, BP is the yield of the better-yielding parent and SC is the yield of the best standard check. The association between GD and hybrid *per se* performance, general combining ability (GCA) and heterosis were estimated using PROC CORR in SAS v9.3 [42].

#### Molecular analysis

Analysis of molecular variance (AMOVA) was performed to estimate the molecular variance components among and within B- and R-line groups using software GenAlEx V6.5 [43] wherein the significance of PhiPT value was tested with 9,999 permutations.

## Results

### Genetic distance, clustering of hybrid parents, and AMOVA

**Fig 2:**
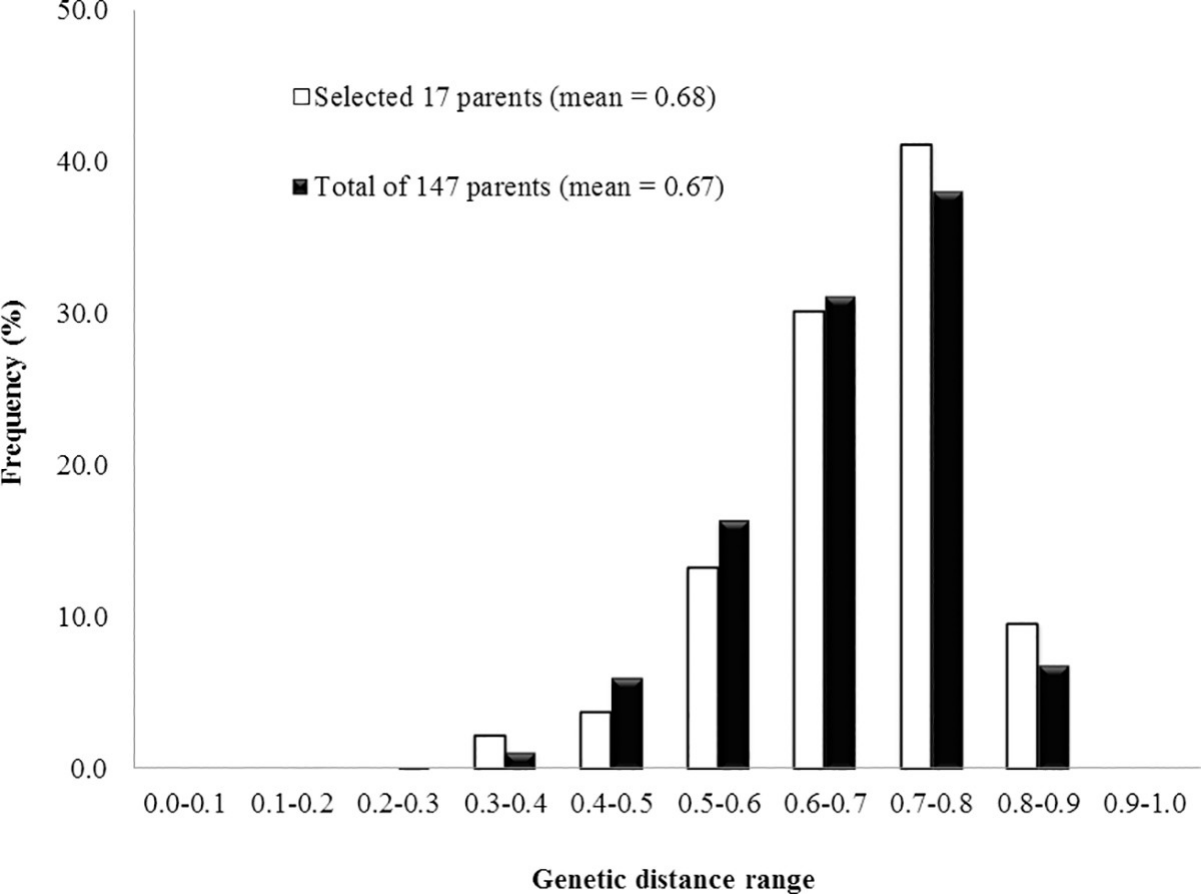
Frequency distribution of genetic distances of all the 147 hybrid parents and for the selected representative set of 17 parents using SSRs.

**Fig 3:**
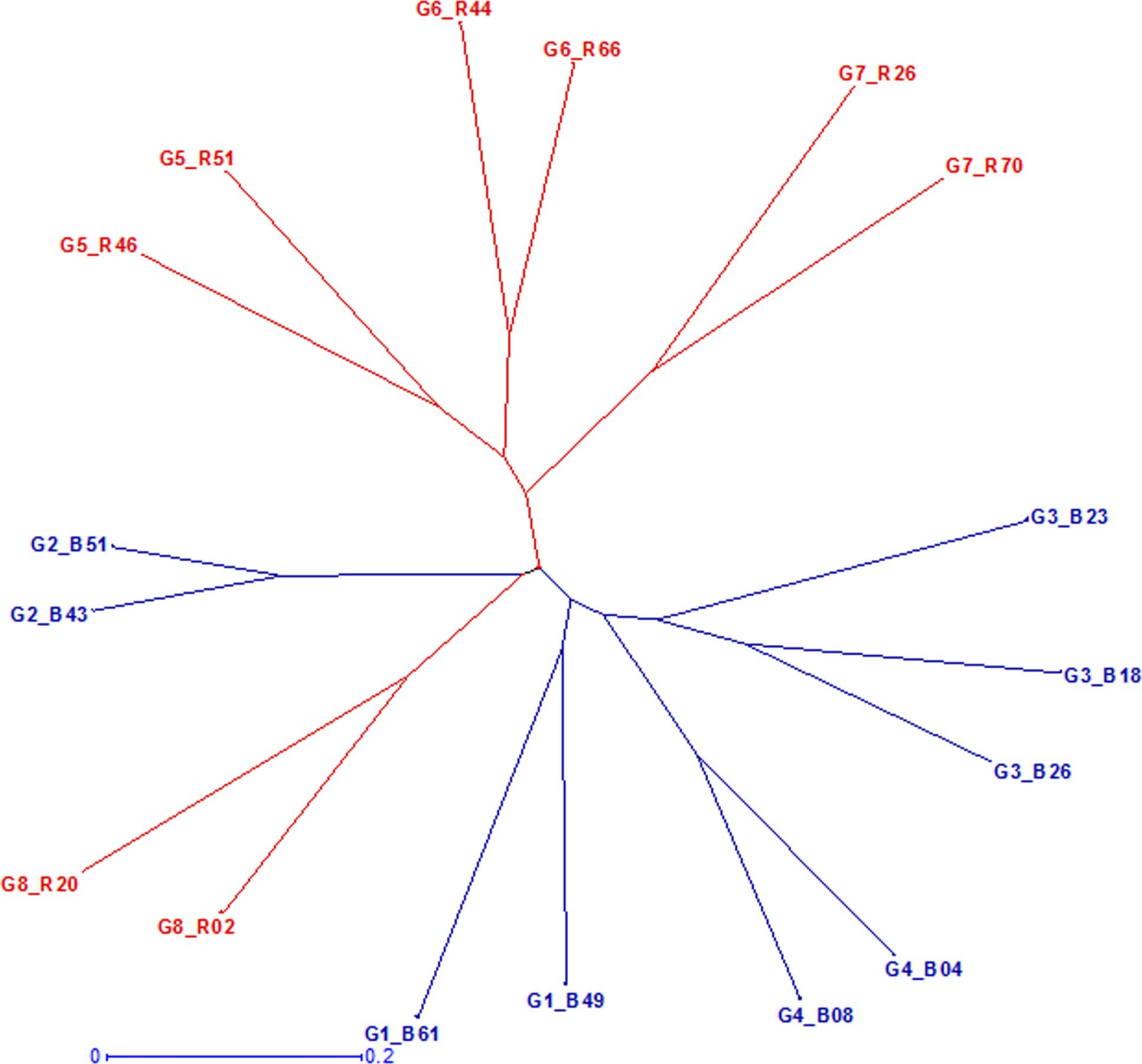
Clustering pattern of selected representative set of 17 hybrid parents using SSRs from the 8 marker based groups (mentioned from G1 to G8).

Fifty six SSRs detected a total of 412 alleles with an average of 7.36 alleles per locus (Table 1) in 147 hybrid parents and all the parents were found distributed across eight clusters (hereafter, mentioned as marker groups and designated as G1 to G8). The mean GD of 17 selected parents was 0.69 with the range of 0.36 to 0.85 (Table 1), which was comparatively similar to the original set of 147 hybrid parents (Table 1) (average GD of 0.68, ranging from 0.17 to 0.90). Also, the frequency distribution of genetic distances between the selected set of representative parents was similar to the distribution as of the GDs of original 147 hybrid parents (Fig 2). Further, a total of 355 alleles were detected in the selected set of the 17 parents which was 86 per cent of the allelic richness present in the original set of 147 hybrid parents (Table 1). The selected hybrid parents clearly grouped into eight marker groups (Fig 3) similar to those as found in the original set of 147 lines (Fig 1). Cluster groups G5, G6, G7 and G8 were dominated by R-lines, while groups G1, G2, G3 and G4 had majority of seed parents (B-lines). The average genetic distance of inter-group pairs was higher than the intra-group pairs (Table 1). Also, B × R pairs had the highest allelic divergence with an average GD of 0.73 followed by R × R and B × B pairs.

**Table 1:** Genetic distance and allelic variation of all the hybrid parents and for the selected representative set of parents using SSRs.

Crosses	Selected parents (17)		All parents (147)	
	Genetic distance^a^	Alleles detected	Genetic distance^a^	Alleles detected
Overall	0.69 (0.36–0.85)	355 (6.33 per locus)	0.68 (0.17–0.90)	412 (7.36 per locus)
B × B pairs	0.60 (0.36–0.77)		0.56 (0.17–0.81)	
R × R pairs	0.67 (0.39–0.79)		0.69 (0.28–0.90)	
B × R pairs	0.73 (0.51–0.85)		0.72 (0.39–0.90)	
Intra-group	0.52 (0.39–0.69)			
Inter-group	0.70 (0.58–0.83)			

^a^ Genetic distance range is given in the parentheses

Results of AMOVA showed highly significant difference between four B-line and four R-line marker based groups (Table 2). The among individuals variation between B- lines or between R-lines was much larger and accounted for 97% and 95% of the total variation, respectively than as observed within individual variation (within B-line or within R-line).

**Table 2:** AMOVA for B- and R-line groups.

Source of variation	Degree of freedom	Sum of squares	Mean sum of squares	Estimated Variance	Variance percentage	P-value	Fst
**B-lines**							
Among Populations	3	186.67	62.22	0.27	1	0.001	0.010
Among Individuals	69	3683.99	53.39	26.44	97		
Within Individuals	73	38.00	0.52	0.52	2		
Total	145	3908.66		27.22	100		
**R-lines**							
Among Populations	3	223.24	74.41	0.60	2	0.001	0.022
Among Individuals	70	3676.54	52.52	25.89	95		
Within Individuals	74	55.50	0.75	0.75	3		
Total	147	3955.28		27.23	100		

### Hybrid performance and combining ability effects

**Fig 4:**
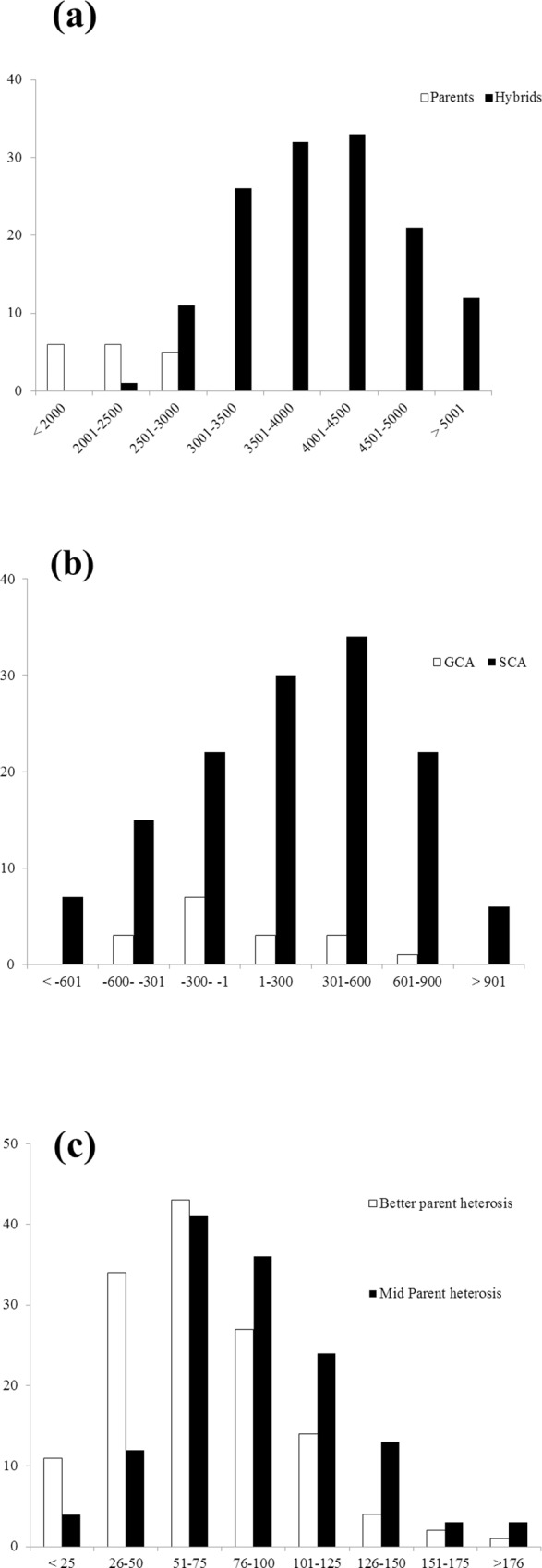
Distribution of (a) grain yield (kg ha^-1^), (b) general and specific combining ability and (c) mid and better parent heterosis, over 136 hybrids.

Analysis of variance revealed presence of significant genetic differences for grain yield in the material under investigation and grain yield was found significantly affected by the environment (Table 3). Large and significant variance due to environment indicated that material was evaluated under diverse environments. Also, the combined ANOVA of 136 hybrids developed using representative lines from eight marker based groups revealed highly significant differences between the groups for grain yield (Table 4). The average grain yield of hybrids (3987 kg ha^-1^) was higher than average grain yield of parents (2167 kg ha^-1^) (Fig 4). A wide range of GCA effects (S4 Table) and specific combining ability (SCA) effects (S4 Table) with significant combining ability variances (S5 Table) indicated the scope of identifying good combiners from this set of parental materials (Fig 4). High positive significant correlation (0.60, p<0.01) was found between GCA and grain yield *per se* of hybrid parents.

**Table 3:** Analysis of variance of hybrids *vis* a *vis* their parents for grain yield in rainy-2015 at two locations (Hisar and Patancheru).

Source of variation	Numerator degree of freedom	Denominator degree of freedom	F-Value	Pr > F
Environment	1	23.5	1342.57	< .0001
Replication (Env)	2	22.2	17.45	< .0001
Genotype	156	248	18.05	< .0001
Hybrid (H)	139	260	14.10	< .0001
Parent (P)	16	273	5.50	< .0001
Hybrid vs Parent	1	24.5	759.54	< .0001
Environment × Genotype	156	248	3.73	< .0001
Environment × Hybrid	139	260	3.91	< .0001
Environment × Parent	16	273	1.46	0.1162
Environment × (H vs P)	1	24.5	15.07	0.0007

**Table 4:** Combined analysis of variance for grain yield of hybrids from eight marker based groups in pearl millet.

Source of variation	Degree of freedom	Sum of squares	Mean sum of squares	P-value	Fst
Between groups	7	6538387.7	934055.4	8.20	< .0001
Within groups	128	14571646.3	113841.0		
Total	135	21110034.0			

Amongst all the 136 hybrids, 55 had significant positive SCA. Of them, 36 hybrids had presence of at least one good general combiner hybrid parent. The hybrid B26 × R11 showed highest significant SCA effect while having H^+^ GCA × H^+^ GCA parental combination, followed by B23 × R66 having L^-^ GCA × H^+^ GCA combination. Maximum numbers of hybrids (16) had L^-^ GCA × H^+^ GCA parental combination. Also, 14 hybrids with significant positive high SCA had both the parents with negative GCA (H^-^/L^-^ GCA × H^-^/L^-^ GCA) for grain yield. Also, MPH ranged from -2.0 to 186.8% with an average of 50.4% while BPH ranged from -13.1 to 175.9% with an average of 32.6% (S6 Table) indicating presence of high heterotic combinations in the material under study. Grain yield of the best check hybrid 86M88 was 5395 kg ha^-1^ and six hybrids had positive yield advantage over the best check 86M88.

### Association between GDs and hybrid performance/heterosis

The GD had significant positive correlation with the hybrid *per se* performance, SCA, BPH and MPH for grain yield (Table 5). Furthermore, to estimate the associations at different levels of GD, all the hybrids in this study were classified into two groups. Sixty eight hybrids having GD in the range of 0.36 to 0.68 were designated as “genetically related”, while rest of the 68 hybrids having GD in the range of 0.69 to 0.85 as “genetically diverse” group. Correlation between GD estimates with both MPH and BPH was moderate in the genetically related group, while it was low in the diverse group. The association results at the group level showed that correlation between the GD and both MPH and BPH was high in B × B crosses while it was low in B × R and R × R crosses.

**Table 5:** Association between SSR based genetic distance and hybrids performance, heterosis and combining ability for grain yield.

Different level of pairs	Number of pairs	Mean *per se* performance for GY	Specific combining ability	Better parent heterosis	Mid parent heterosis
Overall pairs	136	0.27**	0.41**	0.33**	0.37**
Genetically related (0.36 to 0.68 GD)	68	0.40**	0.46**	0.42**	0.41**
Genetically diverse (0.69 to 0.85 GD)	68	0.19	0.16	0.22	0.22
B × B pairs	36	0.42*	0.51**	0.46**	0.47**
B × R pairs	72	-0.01	-0.11	0.21	0.27*
R × R pairs	28	0.04	-0.09	0.03	0.04

*, ** Significant at 0.05, 0.01 levels of probability, respectively

### Heterotic pool formation

B × R crosses had higher mean values for grain yield *per se*, BPH, MPH and SCA estimates than B × B and R × R crosses (Table 6) indicating existence of B- and R- lines as two separate heterotic pools in the available pearl millet hybrid parents. It was also noticed that the inter-group hybrids had higher performance (4008 kg ha^-1^ grain yield, 66.3% BPH, 86.9% MPH and 224.1 units of SCA) than intra-group hybrids (3251 kg ha^-1^ grain yield, 47.8% BPH, 66.2% MPH and -130.9 units of SCA).

**Table 6:** Marker based group wise summary of genetic distance, grain yield, better parent heterosis, mid parent heterosis and specific combining ability for grain yield across the locations.

Fertility crosses	Marker group crosses	Genetic distance	Grain yield (kg ha^-1^)	Better parent heterosis (%)	Mid-parent heterosis (%)	Specific combining ability (Units)
**Inter-group**						
B × R	G1 × G7	0.78	4784.5	83.7	103.1	762.9
B × R	G2 × G7	0.78	4672.9	135.2	154.2	790.0
B × R	G3 × G6	0.76	4546.0	93.8	124.0	263.1
B × R	G1 × G6	0.74	4480.8	89.8	112.1	521.4
B × R	G2 × G6	0.65	4477.9	73.1	92.2	109.1
B × R	G3 × G7	0.83	4466.1	87.4	117.6	509.0
B × R	G3 × G8	0.69	4331.1	58.6	71.1	514.6
B × R	G2 × G8	0.60	4288.6	60.7	71.1	140.0
B × R	G4 × G6	0.78	4234.0	75.7	105.6	487.9
B × R	G1 × G8	0.70	4202.2	57.7	81.5	429.0
B × R	G4 × G7	0.75	3935.3	97.9	124.5	311.3
B × R	G3 × G5	0.70	3905.3	62.0	83.4	517.5
B × R	G1 × G5	0.70	3862.5	84.1	100.8	528.0
B × R	G2 × G5	0.73	3822.6	59.3	80.1	92.7
B × R	G4 × G8	0.72	3706.1	38.9	62.3	196.2
B × R	G4 × G5	0.72	2873.6	35.1	52.5	-193.3
	***B × R***	***0*.*73***	***4185*.*2***	***74*.*4***	***95*.*8***	***396*.*2***
R × R	G6 × G7	0.68	4325.7	92.9	116.3	76.2
R × R	G6 × G8	0.75	4109.4	49.7	66.2	-25.7
R × R	G7 × G8	0.74	4037.7	51.0	84.1	-10.3
R × R	G5 × G8	0.76	3524.9	32.2	55.3	22.0
R × R	G5 × G7	0.70	3495.0	78.4	94.9	-106.2
R × R	G5 × G6	0.62	3459.0	44.8	65.3	-236.5
	***R × R***	***0*.*67***	***3851*.*7***	***60*.*8***	***82*.*6***	***-18*.*1***
B × B	G2 × G3	0.62	4648.1	82.9	94.9	597.7
B × B	G1 × G2	0.62	4207.3	76.8	95.2	186.9
B × B	G2 × G4	0.65	4142.0	69.2	95.2	408.1
B × B	G1 × G4	0.59	3393.5	55.6	76.8	44.8
B × B	G1 × G3	0.61	3311.6	37.9	51.8	-362.8
B × B	G3 × G4	0.58	3298.8	32.9	54.1	-114.2
	***B × B***	***0*.*59***	***3597*.*5***	***50*.*5***	***68*.*0***	***-14*.*0***
**Overall (inter-group)**		**0.70**	**4008.0**	**66.3**	**86.9**	**224.1**
**Intra-group**						
B	G2 × G2	0.60	4356.2	68.5	85.2	-84.6
B	G4 × G4	0.64	3312.1	35.6	73.5	209.1
B	G1 × G1	0.50	3057.1	34.3	55.9	-602.7
B	G3 × G3	0.46	2620.5	0.0	9.2	-1107.6
	***B***	***0*.*55***	***3336*.*5***	***34*.*6***	***56*.*0***	***-396*.*5***
R	G7 × G7	0.69	4456.1	75.9	86.8	745.2
R	G6 × G6	0.39	4256.1	50.8	84.0	-109.5
R	G5 × G5	0.42	3561.4	57.0	87.4	529.4
R	G8 × G8	0.45	3346.0	21.4	25.5	-549.6
	***R***	***0*.*49***	***3904*.*9***	***51*.*3***	***70*.*9***	***153*.*9***
**Overall (intra-group)**		**0.52**	**3251.5**	**47.8**	**66.2**	**-130.9**

**Fig 5:**
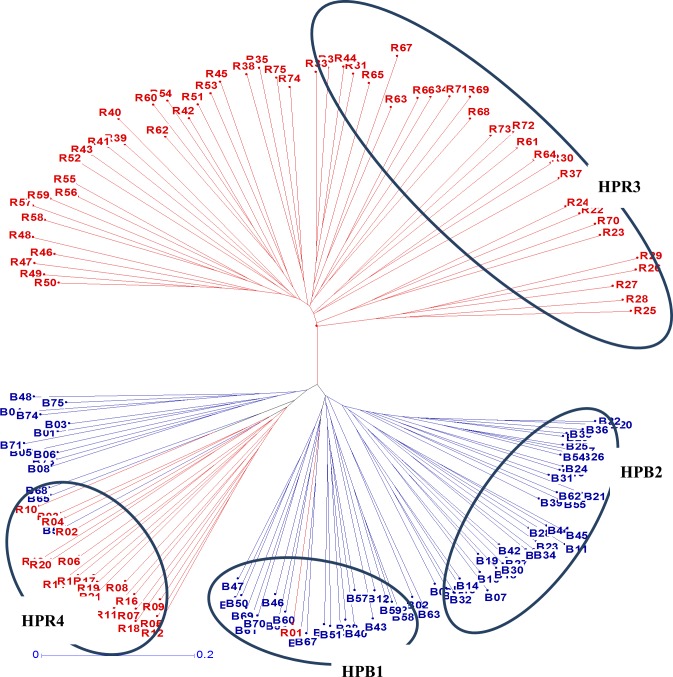
Heterotic pools in the hybrid parents of pearl millet.

In crosses between B- and R-line groups, the hybrids from G1 × G7 had highest average values of grain yield *per se*, BPH, MPH and SCA effects followed by G2 × G7, G3 × G6, G1 × G6 and G2 × G6 (Table 6). The lower yielding hybrids in the B × R groups were from G4 × G5, G4 × G8 and G2 × G5 marker based group crosses; and among them G4 × G5 had the lowest hybrid performance. In general, majority of the best performing B × R group crosses involved lines from G1, G2, G3, G6, G7 and G8 marker based groups (G1, G2, G3 of B-lines; and G6, G7, G8 of R-lines). Lines involving G4 and G5 marker groups had below average grain yield *per se*, comparatively low heterosis and negative combining ability estimates in majority of the cases. Furthermore, G1 or G2 groups of B-lines when crossed with either of G6 or G7 group of R-lines had almost same levels of heterotic parameters (yield *per se*, BPH, MPH and SCA), hence G1 and G2 B-line groups, and G6 and G7 R-line group were merged into common heterotic pools. Therefore, G1+G2 was designated as Heterotic Pool 1 (HPB1); G3 was classified as Heterotic Pool 2 (HPB2), G6+G7 was classified as Heterotic Pool 3 (HPR3), and G8 was classified as Heterotic Pool 4 (HPR4) (Fig 5). HPB1 crossed with HPR3 gave the highest heterosis for grain yield followed by HPB2 × HPR3, HPB1 × HPR4 and HPB2 × HPR4. The highest yielding hybrid was from HPB2 × HPR3 heterotic pool followed by from HPB1 × HPR3 pool.

In crosses within B- and R-line marker groups, the intra-group crosses had lower average grain yield in comparison to inter-group crosses (Table 6). Among inter-group crosses, G6 × G7 in R-lines and G2 × G3 in B-lines had highest yield, while G7 × G7 in R-lines and G2 × G2 in B-lines had the highest yield in intra-group crosses.

## Discussion

This report on heterotic pools in the hybrid parents of pearl millet using molecular markers involved a set of 147 hybrid parents, which represented global diversity available in this crop as majority of them were from ICRISAT’s global pearl millet improvement program. Fifty six SSRs, earlier found distributed across all the linkage groups, showed sufficient polymorphism indicating this to be a highly diverse set of hybrid parents. Clustering pattern formed eight groups which broadly clustered majority of B- and R-lines into two separate clear-cut groups, as shown by AMOVA also, indicating that B- and R-lines exist as two distinct broad-based gene pools. Earlier studies by Nepolean *et al*. [19], Gupta *et al*. [21], and Ramya *et al*. [15] also indicated that B- and R-lines are two separate broad-based gene pools in hybrid parents of pearl millet. However, some of the restorer parents (R-lines) were found closer to seed parents (B-lines) which might be due to occasional involvement of some of trait-specific donor B-line in R-line breeding program, and vice-versa.

One hundred thirty six hybrids developed using representative 17 lines (9 B- and 8 R-lines) by crossing in all possible combinations (excluding reciprocals), evaluated multilocationally along with parents and hybrid checks showed significant variability in parents and hybrids for grain yield, proving that parents selected for this investigation were genetically diverse. A wide range of combining ability and heterosis estimates were recorded for grain yield. A detailed review by Presterl and Weltzien [44] earlier also reported high heterosis for grain yield in pearl millet. Similar results of high combining ability and heterosis in pearl millet were also reported earlier by Vetriventhan *et al*. [45], Jethva *et al*. [46] and Bhadalia *et al*. [47].

The results from all the hybrids suggested low to moderate but significant positive correlation between GD and heterosis/hybrid performance for grain yield. Also, a low positive correlation has been reported earlier between the GD and hybrid grain yield in some other crops, like in maize [48]; rapeseed [49]; wheat [50]; and rice [51]. Contrary to this, Usatov *et al*. [26] in sunflower, reported significant high correlation between marker based GD and grain yield. Although, a review of 54 different studies in several crops on association of GD with hybrid performance by Dias *et al*. [31] revealed that 28 studies showed positive correlation (GD vs hybrid performance), whereas rest of 26 showed negative correlation or inconclusive results.

Few earlier reports have suggested that hybrid performance is not merely a function of genetic distance, but that relationship between heterosis and genetic distance holds true only up to a certain optimum level of genetic distance [4,52–54]. Many researchers have also reported that heterosis can be predicted based on the genetic distance when the parents are genetically related [24,48,55]. Thus, to estimate the association of heterosis at different levels of genetic distance, all the hybrids in this study when classified into two groups *i*.*e*. 50% in “genetically related” (0.37 to 0.68 GDs) and remaining 50% (0.69 to 0.85 GDs) in “genetically diverse” group, showed higher positive correlations (GD vs heterosis) in “genetically related” group than the correlation estimated for “genetically diverse” group. The same result was also evidenced from the correlation between GD and MPH for grain yield in intra- and inter-group hybrids, as correlation was higher in B × B hybrids than in B × R, which might be due to the reason that parents in B × B hybrids were genetically more related than B × R hybrids, which again indicated that correlation (GD vs heterosis) is a function of GD, and it can be positive up to a certain level of genetic distance between parents. Melchinger [16] explained that the presence of same linkage phase for marker and trait loci in the intra-group hybrids might lead to such high correlation in genetically related groups, which could other-wise change in case of the inter-group hybrids so leading to cancellation of positive effect loci by negative ones in genetically diverse groups. Overall, the present study was in general agreement with the conclusion derived by previous studies on the correlation between markers based genetic distance and the hybrid performance/heterosis that the correlation observed in the present study was low to predict heterosis, but could be useful to some extent in predicting heterosis in a group of genetically related individuals.

Pooled mean of MPH and BPH for grain yield of all the B × R crosses (96% and 74% respectively) was higher in comparison to B × B (68% and 50%) and R × R (82% and 61%) crosses indicating B- and R-lines to be existing as two broad-based heterotic pools in pearl millet. The reason for B- and R-lines forming two broad heterotic pools might be due to the way hybrid parents have been bred historically in the pearl millet breeding programs. Most of the seed parents (B-lines) bred at ICRISAT involve use of germplasm from “*togo* origin” (*Togo* is a region comprising of Togo, Ghana, Burkina Faso, and Benin countries of western Africa) having earliness (70–85 days of maturity), relatively less photoperiod-sensitive, large sized grains (15–18 g per 1000) of globular shape and dark grey color, compact and conical panicles; while restorer parents (R-lines) were bred involving germplasm from local (i.e. Indian) adaptation (high tillering, taller height, relatively smaller seed size and profuse pollen production) [56]. Similarly, in some previous studies, broad-based heterotic groups were observed in some other crop breeding programs, like B- and R-line based heterotic groups in rice [12–13]; Reid Yellow Dent (RYD) and Lancaster Sure Crop (LSC) groups in maize [57]; Flint and Dent groups in Maize [58]; and Petkus and Carsten groups in rye (*Secale cereale*) [59].

Further, based on the heterotic pattern shown in this study by groups of B × R hybrids across locations, four heterotic pools *viz*., HPB1 (combining G1 and G2), HPB2 (representing G3), HPR3 (combining G6 and G7) and HPR4 (representing G8) have been identified. All the 25 lines in heterotic pool HPB1 were seed parents (B-lines) and most of them (>50 per cent) had HHVBC (High Head Volume B-lines Composite) and 843B as parents in their pedigrees. In heterotic pool HPB2, all the 35 lines were seed parents and 70 per cent of them shared ICMB 01222 (derived from High Head Volume Composite, with pedigree HHV-S1-24-3-B-3-2-1) as a common parent. In heterotic pool HPR3 all the 31 lines were restorer parents (R-lines) and more than 50 percent of these lines in this pool were found to be bred at the Hisar location, while 23 of the 26 lines in the heterotic pool HPR4 were R-lines. Among these heterotic pools, HPB1 x HPR3 demonstrated the highest level of average *per se* performance and heterosis followed by HPB2 x HPR3 as compared to the mean of all hybrids *per se* performance and their heterosis for grain yield. As HPB1 and HPB2 comprised the seed parents bred at Patancheru (mostly having *togo* origin) while majority of the inbred lines in HPR3 belongs to Indian program (Hisar center) indicating that *togo* origin seed parents when crossed with locally adapted restorer parents gave highest levels of heterosis. Parents of six hybrids having positive significant standard heterosis (heterobeltiosis) for grain yield outperformed the highest yielding hybrid 86M88 (leading pearl millet hybrid in India, bred by Pioneer Overseas Corporation), and the parental lines of these hybrids belonged to heterotic pools HPB1, HPB2 and HPR3.

Among the 55 hybrids having high significant positive SCA effects, nine having H^+^ GCA × H^+^ GCA parent combinations indicating additive genetic component or additive × additive interaction to be responsible for the high heterosis. While, eight crosses with H^+^ GCA × H^-^ GCA, 3 with L^+^ GCA × H^+^ GCA and 16 with H^+^ GCA × L^-^ GCA revealed both additive and non-additive genetic components to be responsible for the high heterosis. Furthermore, the superiority of the 14 crosses having both the parents with low GCA showed their specific gene combinations resulted in high SCA effects. Therefore, results suggested that both GCA and SCA effects are important for expression of heterosis and should be precisely tested with the appropriate testers in any hybrid breeding program. An important observation in this study was that the GCA for grain yield was found to be positively correlated with *per se* performance of hybrid parents indicating that high general combiners are more likely to occur in lines having high grain yield. Hence, high *per se* yield of parental lines, in addition to lowering the seed cost, could be used as an indicator of good combining ability. A similar positive correlation was reported earlier by Rai and Virk [60] in pearl millet.

Overall, the results of this study suggested that molecular markers based genetic distance have potential to predict heterosis up to a certain level of genetic distance between the parents. The study was able to identify B- and R-lines as two separate broad heterotic groups in pearl millet and suggested four heterotic pools, two each of seed (B-lines) and restorer (R-lines) parents, which will help future pearl millet breeders not only in developing high yielding hybrids but also in the development of highly productive hybrid parents for elevating the potential of hybrid technology in this crop.

## Supporting information

S1 Table Name (code) and pedigrees of pearl millet parental lines as found in different marker based groups. (DOCX)

S2 Table Information about primer sequence and linkage group of SSR markers used in the present study. (DOCX)

S3 Table Distribution of 147 pearl millet hybrid parents in different groups based on SSR genotyping, size of groups and number and name of identified representative parents. (DOCX)

S4 Table GCA and SCA of the pearl millet parental lines and hybrids with grain yield of representative hybrid parental lines involved in this study. (DOCX)

S5 Table Analysis of variance for combining ability. (DOCX)

S6 Table Better-parent heterosis (above diagonal) and mid-parent heterosis (below diagonal) of pearl millet hybrids. (DOCX)
