# Supplementary material for: Formation of heterotic pools and understanding relationship between molecular divergence and heterosis in pearl millet [Pennisetum glaucum (L.) R. Br.]
Source: PLoS One. 2019 May 7;14(5):e0207463. doi: 10.1371/journal.pone.0207463 (PMC6504090; doi:10.1371/journal.pone.0207463)
Supplement: S1 Table — (DOCX) [file pone.0207463.s001.docx]

**S1 Table. Name (code) and pedigrees of pearl millet parental lines as found in different marker based groups.**

| Marker based groups | Codes | Pedigree |
| --- | --- | --- |
| G1 | B-46 | (HHVDBC HS-246-1-2-1-3 x ICMB 98444)-4-3-4-4 |
| G1 | B-47 | [[{ICMV 88908-11-12-3-2-B x B-bulk)-8-B-3 x {(843B x ICMPS 900-9-3-2-2)-41-2-5-5 S2-34-1-2-1-1 x B-bulk}-5-B-B]-11-1-1-B-B x ICMB 04111]-67-5-2-1 |
| G1 | B-49 | [[{ICMV 88908-11-12-3-2-B x B-bulk)-8-B-3 x {(843B x ICMPS 900-9-3-2-2)-41-2-5-5 S2-34-1-2-1-1 x B-bulk}-5-B-B]-11-1-1-B-B x ICMB 04111]-69-2-1-1 |
| G1 | B-50 | [[{ICMV 88908-11-12-3-2-B x B-bulk)-8-B-3 x {(843B x ICMPS 900-9-3-2-2)-41-2-5-5 S2-34-1-2-1-1 x B-bulk}-5-B-B]-11-1-1-B-B x ICMB 04111]-69-2-3-1 |
| G1 | B-53 | [(ICMR 312 S1-1-5-2-B x HHVBC)-10-2-1-2-3 x EEBC 407)-7-2-1-3 |
| G1 | B-60 | [(ICMR 312 S1-1-5-2-B x HHVBC)-10-2-1-2-3-B x HHVDBC HS-158-2-1-2-1-1-B]-1-1-3-1 |
| G1 | B-61 | HMS 6B |
| G1 | B-66 | HMS 28B |
| G1 | B-67 | HMS 29B |
| G1 | B-69 | HMS 33B |
| G1 | B-70 | HMS 36B |
| G1 | R-1 | (AIMP 92901 S1-480-1-1-1-2-B-2 x ICMR 312 S1-3-2-3-2-1-1-B-B)-B-9-2-1-B |
| G2 | B-2 | Togo-11-5-2 selection |
| G2 | B-12 | {HHVDBC HS-10-1-2-1-1-4 x [ICMB 99555 x {78-7088/3/SER3 AD//B282/(3/4)EB x PBLN/S95-359}-19-5-B-B]-13-2-B-B-B}-22-3-2-2-1 |
| G2 | B-37 | [ICMB 95111 x (D2BLN/95-107 x EEBC C1-1)-6-B]-24-4-1-B-B-B-B-11-1] x B-bulk (3981-3989/S06 G1)}-2-3-1-B |
| G2 | B-38 | (EEDBC S1-425-2-1-2-3-B-1-B-8-1 x B-bulk (3981-4011/S06 G1))-3-3-3-B |
| G2 | B-40 | EEDBC S1-2-1-1-1-1-1-1-B-B-2-3-B-B |
| G2 | B-41 | {[(843B x ICTP 8202-161-5)-20-3-B-B-3 x B-bulk]-2-B-1-2-2-B-B-B-11-1 x B-bulk (3981-4011/S06 G1)}-3-2-4-4 |
| G2 | B-43 | (ICMB 04888 x ICMB 02333)-3-1-3-1 |
| G2 | B-51 | {[(843B x ICTP 8202-161-5)-20-3-B-B-3 x B-bulk]-2-B-1-2-2-B-B-B-11-1 x B-bulk (3981-4011/S06 G1)}-3-3-4 |
| G2 | B-52 | {(MC 94 S1-34-1-B x HHVBC)-16-2-1-1-1-1-B-B-5 x (MC 94 S1-34-1-B x HHVBC)-10-4-1-2-1-B-B-1-30-2-4-3-3 |
| G2 | B-57 | (843B x EEBC S1-407)-12-3-B |
| G2 | B-58 | (ICMB 01666 x ICMB 01222)-49-1-2-B-2 |
| G2 | B-59 | [(MC 94 S1-34-1-B x HHVBC)-10-4-3-2-2-B-B-2 x (ICMR 312 S1-1-5-3-B x HHVBC)-7-1-1-1-B-B-B]-21-B-1-4-1-2-1-3 |
| G2 | B-63 | HMS 14B |
| G3 | B-7 | [HHV-S1-24-3-B-3-2 x (ICMB 91777 x HHVBC)]-5-B-1-1-B-B-B |
| G3 | B-9 | [ICMB 97444 x (D2BLN/95-98 x EEBC C1-1)-7-B-B]-34-2-4-B-B-5-B-B |
| G3 | B-10 | [78-7088/3/SER3 AD//B282/(3/4)EB x PBLN/S95-359]-7-4-B-B-6-1-B-1-B-1-B-B-B |
| G3 | B-11 | [(ICMB 95111 x 9035/S92-B-3)-17-5-1-B-B-B x ICMB 99111]-3-2-1-3 |
| G3 | B-13 | [(D2BLN/95-93 x SPF1/K95-3213-20)-10 x (91777B x HHVBC)]-7-B-1-B-B-4-B-B-B-B-2 |
| G3 | B-14 | (EEDBC S1-425-2-1-2-3-B-1-B-7-1 x B-bulk (3981-4011/S06 G1))-2-2-3 |
| G3 | B-15 | IC-CZBC-C0-166-2-1-3-B |
| G3 | B-16 | (ICMB 01888 x ICMB 01222)-16-1-2-2-1-B-B |
| G3 | B-17 | (ICMB 93333 x ICMB 01222)-11-1-B-7-1 |
| G3 | B-18 | (ICMB 01888 x ICMB 01222)-1-2-B-5-2 |
| G3 | B-19 | (ICMB 99555 x ICMB 00555)-5-4-3-B-B-2-3-2 |
| G3 | B-20 | (ICMB 99555 x ICMB 00555)-5-4-3-B-B-3 |
| G3 | B-21 | [(843B x ICTP 8202-161-5)-20-3-B-B-3 x B-bulk]-2-B-2-B-1-3 |
| G3 | B-22 | (ICMB 01666 x ICMB 01222)-49-1-2-7-B |
| G3 | B-23 | [HHV-S1-24-3-B-3-2 x (ICMB 96333 x HHVBC)]-19-B-1-3-B-B-B-B |
| G3 | B-24 | HHVDBC HS-221-1-4-1-1-1-B-2 |
| G3 | B-25 | (ICMB 93333 x ICMB 01222)-11-2-2-5-B-B |
| G3 | B-26 | (ICMB 93333 x ICMB 01222)-11-2-2-2-B-2-B |
| G3 | B-27 | [(843B x ICMPS 900-9-3-2-2)-41-2-5-5 S2-34-1-2-3 x B-bulk]-8-B-B-1-B-B-B-2-B |
| G3 | B-28 | [(ICMB 95111 x 9035/S92-B-3)-17-5-1-B-B-B x ICMB 99111]-3-2-1-2 |
| G3 | B-29 | (ICMB 01888 x ICMB 01222)-16-1-2-3-3-4 |
| G3 | B-30 | (HTBLN/95-98 x ICMB 89111)-8-B-B-B-B-2-B |
| G3 | B-31 | [HHV-S1-24-3-B-3-2 x (ICMB 91777 x HHVBC)]-12-B-3-1-B-B-2 |
| G3 | B-32 | NC D2 S1-17-2-1-1-2-2-B-4 |
| G3 | B-33 | (ICMB 97444 x ICMB 00888)-52-1-1-B-4-2-B |
| G3 | B-34 | (ICMB 97444 x ICMB 00888)-52-1-1-B-4-3-B |
| G3 | B-35 | (ICMB 01888 x ICMB 01222)-16-1-2-3-2-3-B |
| G3 | B-36 | (ICMB 93333 x ICMB 01222)-20-2-B-5-B-1-B |
| G3 | B-39 | IC-CZBC-C0-46-3-3-1-2 |
| G3 | B-42 | (ICMB 04888 x ICMB 00444)-7-1-3-2 |
| G3 | B-44 | [(ICMB 95111 x 9035/S92-B-3)-17-5-1-B-B-B x ICMB 99111]-3-2-4-B |
| G3 | B-45 | [(ICMB 95111 x 9035/S92-B-3)-17-5-1-B-B-B x ICMB 99111]-11-1-1-B |
| G3 | B-54 | (ICMB 93333 x ICMB 01222)-20-2-B-5-B-4-B |
| G3 | B-55 | (ICMB 01888 x ICMB 01222)-16-1-2-3-5-B-B |
| G3 | B-62 | HMS 7B 1 |
| G4 | B-1 | Selection from KSU line BKM 2068 |
| G4 | B-3 | [843B x (J 1623 x 3/4 EB-96-1-10)]-5-2 |
| G4 | B-4 | (EBC-S1-40-2-2-1 x B-bulk)-25-B-B |
| G4 | B-5 | (ICMB 95444 x ICMB 93333)-24-2-B-B |
| G4 | B-6 | (ICMB 95444 x ICMB 92111)-4-B-4-3-B-B |
| G4 | B-8 | (ICMB 95444 x ICMB 93333)-24-2-B-4-B-B-B |
| G4 | B-48 | (B x B) F2 (G-4)-72-4-3-3 |
| G4 | B-71 | HMS 37B |
| G4 | B-73 | HMS 40B |
| G4 | B-74 | HMS 42B |
| G4 | B-75 | HMS 50B |
| G5 | R-39 | ICMS 7704-S1-52-3-1-2-1-2-1-6-B-B |
| G5 | R-40 | IP No. 9348-1-2-1-1 |
| G5 | R-41 | MC 94 C2-S1-3-1-3-3-2-2-B |
| G5 | R-42 | MRC S1-340-1-3-3-2-B-B-1 |
| G5 | R-43 | RCB-2 S1-19-2-5-1-1-2-3-3-B-B-B-B-B |
| G5 | R-45 | MRC HS-130-2-2-1-B-B-1-B-B-B-B-1-B-B |
| G5 | R-46 | MRC HS-170-3-5-2-B-B-2-B-B-B-1 |
| G5 | R-47 | MRC HS-225-3-5-2-B-B-B-1 |
| G5 | R-48 | MRC HS-91-2-3-3-B-B-B-8-B-B-B-B-1-B |
| G5 | R-49 | MRC S1-155-4-3-B-B-B-B-1-B-B |
| G5 | R-50 | MRC S1-155-4-3-B-B-B-B-1-B-B-1 |
| G5 | R-51 | MRC S1-9-1-1-B-B-B-B-B-B (ISK sel)-1 |
| G5 | R-52 | RCB-2 S1-19-2-5-3-2-3-1-1-B-1-1-B |
| G5 | R-53 | RIB 3135/18 |
| G5 | R-54 | MRC HS-170-3-5-2-B-B-2-B-B-B-1 |
| G5 | R-55 | (EERC-HS-23)-3-1-4 |
| G5 | R-56 | (EERC-HS-29)-B-12-4-1-1-B-B |
| G5 | R-57 | (EERC-HS-6)-B-12-1-1-3-B |
| G5 | R-58 | (EERC-HS-8)-B-5-1-1-1 |
| G5 | R-59 | (MC 94 C2-S1-3-2-2-2-1-3-B-B x AIMP 92901 S1-488-2-1-1-4-B-B)-B-25-4-2-3-B-B |
| G5 | R-60 | (MRC HS-86-1-1-5-B-B-B-B-B x MRC S1-9-2-2-B-B-2-B-B)-10 |
| G6 | R-31 | [((MC 94 S1-34-1-B x HHVBC)-16-2-1) × (IP 19626-4-2-3)]-B-34-1-3-3-1-1-B-2 |
| G6 | R-32 | [IP 12370-1-3 × B-Lines]-B-14-1-1-2-4-1-1-B |
| G6 | R-33 | {[((MC 94 S1-34-1-B x HHVBC)-16-2-1) × (IP 19626-4-2-3)]-B-28-3-3-3-2}×{MRC HS 225-3-5-2-B-B-B-B}-B-1-3-4-1-B |
| G6 | R-34 | Acid tolerant pop S1-7-1-5-2-B |
| G6 | R-35 | AIMP 92901 S1-296-2-1-1-4-2-B-12-5-1-1-B |
| G6 | R-38 | ICMR 312 S1-8-1-1-1-1-B-B-B-1-B |
| G6 | R-44 | LaGrap C2-S1-38-2-1-1-1-B-B |
| G6 | R-63 | RAJ 3 |
| G6 | R-65 | TCP-10-110 |
| G6 | R-66 | HPT-10-129 |
| G6 | R-67 | TCH 26-1 |
| G6 | R-68 | SGP-10-120 |
| G6 | R-69 | HPT-10-144 |
| G6 | R-71 | TCP-10-124 |
| G6 | R-74 | TPT-A2-1-11-155 |
| G6 | R-75 | PT-1-10-1131 |
| G7 | R-22 | JBV 3 S1-166-3-1-1-1-1 |
| G7 | R-23 | MRC HS-86-1-1-2-B-B-2-B-B-1 xR-lines bulk pollen (20275-20291) |
| G7 | R-24 | MRC S1-156-1-1-1-B-3-B-B-B-8-1-1-B |
| G7 | R-25 | MRC S1-9-2-2-B-B-2-B-B-1-B-B |
| G7 | R-26 | (EERC-HS-8)-B-5-1-1-3 |
| G7 | R-27 | (J 834 x 700516)-1-4-4-2-4-B-2-2-B-B-B-1-B-B |
| G7 | R-28 | (JBV 3 S1-197-1-1-1 x IPC 655(B))-B-2-1 |
| G7 | R-29 | (MC 94 C2-S1-3-1-3-3-1-2-1 x SDMV 90031 S1-3-3-2-2-2-2-2)-B-8-2-1 |
| G7 | R-30 | (MC 94 C2-S1-3-2-2-2-1-3-B-B x ICMR 312 S1-3-2-3-2-1-1-B-B)-B-23-2-1 |
| G7 | R-37 | IAC-ISC TCP5 S1-2-1-1-B-1-2-B-4-B |
| G7 | R-61 | HTP 94 / 54 |
| G7 | R-64 | HFIT-3-11-125 |
| G7 | R-70 | PT-1-10-1047 |
| G7 | R-72 | H77/833-2-202 |
| G7 | R-73 | SGP-10-107 |
| G8 | B-56 | [(ICMB 96555 x IP 10437)-3-4-1-2-3x{(96555B x LaGrap C2 S1-32-1)-10}xIP 14758-2-1]-2-B-1-3-1-3 |
| G8 | B-65 | HMS 26B |
| G8 | B-68 | HMS 30B |
| G8 | R-2 | (EERC-HS-7)-B-6-4-3-2-B-B |
| G8 | R-3 | (ICMV-IS 94206-7 × (SRC II C3 S1-1-1-2 x HHVBC)-1-3-3))-B-10-1-1-5-4-1-2 |
| G8 | R-4 | (IPC 107 ×ICMV 91059 S1-14-2-1-1-2)-13-2-2-2-1-B |
| G8 | R-5 | (IPC 1268 ×ICMV 91059 S1-58-2-2-2-1)-8-1-1-7-B-B-B |
| G8 | R-6 | (IPC 1617 ×SDMV 90031-S1-84-1-1-1-1)-26-2-3-1 |
| G8 | R-7 | [(((ICMV-IS 94206-15)×B-Lines)-B-6) × (MRC S1-405-1-2-B)]-B-4-1-1-2-B-5-B |
| G8 | R-8 | [(((IP 12322-1-2)×B-Lines)-B-8) × (MRC S1-155-4-2-B)]-B-14-2-2-1-1-B-4 |
| G8 | R-9 | [(AIMP 92901 S1-488-2-1-1-2-B-1-B x R-lines bulk 20216-20249/K09)]-13-3 |
| G8 | R-10 | [(IPC 107×SDMV 90031-S1-84-1-1-1-1)×AIMP 92901 S1-296-2-1-1-3-B-1]-3-2-2-2-1-B-5 |
| G8 | R-11 | [(IPC 1268×ICMV 91059 S1-58-2-2-2-1)×AIMP 92901 S1-296-2-1-1-1-B-B]-2-2-1-2-4-B |
| G8 | R-12 | [(IPC 1617×SDMV 90031-S1-84-1-1-1-1)×AIMP 92901 S1-296-2-1-1-3-B-1]-4-4-5-3-2-B-B |
| G8 | R-13 | [(IPC 337×SDMV 90031-S1-84-1-1-1-1)×ICMS 8511 S1-14-3-1-1-2-B-1]-3-2-4-4-1-B-B |
| G8 | R-14 | [(IPC 337×SDMV 90031-S1-84-1-1-1-1)×RCB-2-S1-144-2-2-2-1-1-1]-1-1-3-1-2-B-B |
| G8 | R-15 | [(IPC 337×SDMV 90031-S1-84-1-1-1-1)×SDMV 95045 S1-7-2-4-2-3-2-2-2-1]-P1-1-4 |
| G8 | R-16 | [(RCB-2 S1-19-2-5-1-1-2-3-3-B-B-B-B-Bx(MC 94 C2-S1-3-2-2-2-1-3-B-B x AIMP 92901 S1-488-2-1-1-4-B-B)-B-8-3-1)]-7 |
| G8 | R-17 | {(SRC II C3 S1-19-3-2 X HHVBC)-1-5-1} X {[((96111b X 4017-6-1-1)-1-4-4-3) X (IP 19626-4-1-2-1)]-B-6}-B-5-1-1-3 |
| G8 | R-18 | {[((MC 94 S1-34-1-B x HHVBC)-16-2-1) × (IP 19626-4-2-3)]-B-18-2-3-2-2-BxAIMP 92901 S1-296-2-1-1-3-B-1-6-B-B}-B-11-1 x {[((MC 94 S1-34-1-B x HHVBC)-16-2-1) × (IP 19626-4-2-3)]-B-28-2-2-3-1-2xICMR 312 S1-3-2-3-2-1-1-B-B-B-B}-B-14-3 |
| G8 | R-19 | HHVBC tall (C1) S1-33-3-1-1-1-2-B-B-3-2-B |
| G8 | R-20 | ICMS 8506 S1-4-2-2-2-3-3-1-2-3-1-B-3 |
| G8 | R-21 | ICMV 91059 S1-20-1-2-2-3-2-B-1-1-2-B-5-2-1-B |
|  | B-64 | HMS 22B |
|  | B-72 | HMS 39B |
|  | R-36 | GB 8735-S1-15-3-1-1-3-4-2-2-1-1-B-B |
