# Supplementary material for: Formation of heterotic pools and understanding relationship between molecular divergence and heterosis in pearl millet [Pennisetum glaucum (L.) R. Br.]
Source: PLoS One. 2019 May 7;14(5):e0207463. doi: 10.1371/journal.pone.0207463 (PMC6504090; doi:10.1371/journal.pone.0207463)
Supplement: S2 Table — (DOCX) [file pone.0207463.s002.docx]

**S2 Table. Information about primer sequence and linkage group of SSR markers used in the present study**

| Markers | LG | Repeat Motif | Primer sequence | | References |
| --- | --- | --- | --- | --- | --- |
| *Xctm08* | 7 | (CT)8(CT)11 | F  R | GCTGCATCGGAGATAGGGAA  CTCAGCAAGCACGCTGCTCT | Budak *et al*., 2003 |
| *Xctm10* | 3 | (CT)22 | F  R | GAGGCAAAAGTGGAAGACAG  TTGATTCCCGGTTCTATCGA | Budak *et al*., 2003 |
| *Xctm12* | 1 | (CT)12 | F  R | GTTGCAAGCAGGAGTAGATCGA  CGCTCTGTAGGTTGAACTCCTT | Budak *et al*., 2003 |
| *Xicmp3002* | 6 | (AAG)7 | F  R | AAGATGGATGATGGATTGATGA  T ACACACACATTGCCACACG | Senthilvel *et al*., 2008 |
| *Xicmp3032* | 1 | (GCT)8 | F  R | GCGTAGACGGCGTAGATGAT  CAACAGCATCAAGCAGGAGA | Senthilvel *et al*., 2008 |
| *Xicmp3043* | 7 | (AGC)5 | F  R | TCCTGTACAAGGACGTGCAG  TATCGACGCCAACGATACTG | Senthilvel *et al*., 2008 |
| *Xicmp3048* | 7 | (GTGCG)3 | F  R | CGGAACTGCTGGAGTGAAAT  GCGACTTCGACCGACTTTT | Senthilvel *et al*., 2008 |
| *Xicmp3080* | 1 | (AGC)8 | F  R | CAAACAGCATCAAGCAGGAG  GCGTAGACGGCGTAGATGAT | Senthilvel *et al*., 2008 |
| *Xicmp3088* | 1 | (TCC)8(TCTA)4 | F  R | TCAGGTGGAGATCGATGTTG  TTACGGGAGGATGAGGATG | Senthilvel *et al*., 2008 |
| *Xipes0004* | 1 | (GT)11 | F  R | GTGCGTTCTTCCTTGCCTAC  TCATCACACAGGGCTAGCTG | Rajaram *et al*., 2013 |
| *Xipes0082* | 7 | (AGGAG)7 | F  R | CGACCCCTGAAGGAAATCTT  TTCTTCATGTGGGTGTCGAA | Rajaram *et al*., 2013 |
| *Xipes0105* | 7 | (GGTTG)5 | F  R | GGGGGCTCACAGAACAAGTA  CCGAAGTTCCCACAGAATGT | Rajaram *et al*., 2013 |
| *Xipes0152.2* | 5 |  | F  R | TACGAAGGGAAGCACAGC  TGTGTGGTAAGCTGCTGGAG | Rajaram *et al*., 2013 |
| *Xipes0174* | 4 | (CCGT)5 | F  R | TCTGGGAAGGAGGAGGATTT  TGCTGCTGCTCTCTGACTGT | Rajaram *et al*., 2013 |
| *Xipes0176* | 6 | (TGC)7 | F  R | TGGTGCAAGAATGACCATGT  CGCAGGATTACAAACATCCA | Rajaram *et al*., 2013 |
| *Xipes0186* | 4 | (TTG)10 | F  R | AGCATATGGCATCCTTTTCG  TTTCAGGCTTGGATTCAATGT | Rajaram *et al*., 2013 |
| *Xipes0198* | 7 | (AATACC)8 | F  R | GGGGAGCTCTCTCTGAACTG  GAACCGCTTCTTCATCCATC | Rajaram *et al*., 2013 |
| *Xipes0200* | 6 | (GTAC)11 | F  R | GCGCTTTCAGAGTCCTGAGT  CAAGTCGTCACGGCCTTATT | Rajaram *et al*., 2013 |
| *Xipes0203* | 1 | (ATC)16 | F  R | CCCTCGAAGAGATCGAAGTG  CTGAAACAACAGCCTGCAAA | Rajaram *et al*., 2013 |
| *Xipes0213* | 3 | (GAT)4 | F  R | GTCCGGTTTGTCTCTCCTTG  TGGATCTCCCATGTCGTGTA | Rajaram *et al*., 2013 |
| *Xipes0220.1* | 3 |  | F  R | CGTGGTCGATGGACTGCT  CGAGAGATTCACACCAAGCA | Rajaram *et al*., 2013 |
| *Xipes0223* | 5 | (AT)7 | F  R | ATTTCCTTGGGATTGTGCAG  ATTTCCTTTCCCCAAACGAC | Rajaram *et al*., 2013 |
| *Xipes0227* | 6 | (GAT)4 | F  R | GCTGAGGTGGAGAAGTTTGC  GGGGGTGGTTATGAGCCTAT | Rajaram *et al*., 2013 |
| *Xipes0236* | 2 | (TGG)11 | F  R | GGCCAGCTCGCCTAGAT  AGATCCACCGCCTAATGAAA | Rajaram *et al*., 2013 |
| *Xpsmp2045* |  | (CA)11(GA)5 | F  R | TCATCTTCCCCTATCCGAAAC  ACTTGCCAATGCTATCTTCAC | Qi *et al*., 2004 |
| *Xpsmp2059* | 2 | (AC)11 | F  R | GGGGAGATGAGAAAACACAATCAC  TCGAGAGAGGAACCTGATCCTAA | Qi *et al*., 2004 |
| *Xpsmp2068* |  | (AC)14 | F  R | CAATAACCAAACAAGCAGGCAG  CTTCACTCCCACCCTTTCTAATTC | Qi *et al*., 2004 |
| *Xpsmp2070* | 3 | (CA)25(TA)6 | F  R | ACAGAAAAAGAGAGGCACAGGAGA  GCCACTCGATGGAAATGTGAAA | Qi *et al*., 2004 |
| *Xpsmp2077* | 2 | (CA)15(TA)8 | F  R | GCCAATATTATTCCCAAGTGAACA  CTCTTGGTTGCATATCTTTCTTTT | Qi *et al*., 2004 |
| *Xpsmp2079.2* | 7 |  | F  R | CAGCCGAAGGCTAATCAACAA  GTGGTCAGCAGCAGATGTAA | Nepolean *et al*., 2012 |
| *Xpsmp2081.1* | 4 |  | F  R | CCTGTGCTGTCATTGTTACCA  TCAGATCACCTATTACTTTCCCT | Rajaram *et al*., 2013 |
| *Xpsmp2086* | 4 | (AC)14 | F  R | CGCTTGTTTTCCTTTCTTGCTGTT  CCTTCTCAGATCCTGTGCTTTCTT | Qi *et al*., 2004 |
| *Xpsmp2089* | 2 | (AC)15 | F  R | TTCGCCGCTGCTACATACTT  TGTGCATGTTGCTGGTCATT | Qi *et al*., 2004 |
| *Xpsmp2090* | 1 | (CT)12) | F  R | AGCAGCCCAGTAATACCTCAGCTC  AGCCCTAGCGCACAACACAAACTC | Qi *et al*., 2004 |
| *Xpsmp2201* | 2 | (GT)6 | F  R | CCCGACGTTATGCGTTAAGTT  TCCATCCATCCATTAATCCACA | Qi *et al*., 2001 |
| *Xpsmp2202* | 5 | (GT)8 | F  R | CTGCCTGTTGAGAATAAATGAG  GTTCCGAATATAGAGCCCAAG | Qi *et al*., 2001 |
| *Xpsmp2203* | 7 | (GT)18imperfect | F  R | GAACTTGATGAGTGCCACTAGC  TTGTGTAGGGAGCAACCTTGAT | Qi *et al*., 2001 |
| *Xpsmp2204* |  | (GT)7 | F  R | TGCTTCTTGACTATGTTTTCC  AGATATGGCGAACGTGAGGAG | Qi *et al*., 2001 |
| *Xpsmp2207* |  | (GT)5 | F  R | CAGGGCATACTTCAAGATTGATTC  GTCCACTTGTTATTCTCTATCACC | Qi *et al*., 2001 |
| *Xpsmp2208* | 5 | (GT)10 | F  R | GAAAGAGCAAACTGAACAATCCC  ACTTTGCCCTGGATGATCCTC | Qi *et al*., 2001 |
| *Xpsmp2209* |  | (GT)6(CT)7 | F  R | TTGGACGATTTGGAAGCATAG  GAGGAAAAGAGCCATACAGAGAC | Qi *et al*., 2001 |
| *Xpsmp2211* | 2 | (GT)6 | F  R | CTGCATGACGTGTGACCAATACC  AACAAATCAGCACCAGCCTCC | Qi *et al*., 2001 |
| *Xpsmp2212* |  | (GT)6 | F  R | GATTGGATGGCAGTGCTTGG  CAAACCAGCCATCAACAACCAG | Qi *et al*., 2001 |
| *Xpsmp2214* | 3 | (GT)9 | F  R | CGCACAGTACGTGTGAGTGAAG  GATTGAGCAGCAAAAACCAGC | Qi *et al*., 2001 |
| *Xpsmp2218* |  | (GT)12(GA)30 | F  R | CTCTGTAAGTTCCTGGTGCTCAA  TCAGGCCAGTAACACATCTCAA | Qi *et al*., 2001 |
| *Xpsmp2220* | 5 | (GT)11 | F  R | GCATCCTTCACCATTCAAGACA  TGGGAAACAGAATGGAGAAAAGAG | Qi *et al*., 2001 |
| *Xpsmp2222* |  | (GT)6 | F  R | TGGCTTCCAGACTAATCATCAC  TTATTTTAGCGGCGAGATTGAC | Qi *et al*., 2001 |
| *Xpsmp2227* | 3 | (GT)7 | F  R | ACACCAAACACCAACCATAAAG  TCGTCAGCAATCACTAATGACC | Allouis *et al*.,2001 |
| *Xpsmp2232* | 1 & 2 | (TG)8 | F  R | TGTTGTTGGGAGAGGGTATGAG  CTCTCGCCATTCTTCAAGTTCA | Allouis *et al*.,2001 |
| *Xpsmp2237* | 2 | (GT)8 | F  R | TGGCCTTGGCCTTTCCACGCTT  CAATCAGTCCGTAGTCCACACCCCA | Allouis *et al*.,2001 |
| *Xpsmp2246* | 1 | (TG)7imperfect | F  R | CGGATGCTAAATTAACCGAAGC  CCAGCTTGCTTCTGTTGCGTTC | Allouis *et al*.,2001 |
| *Xpsmp2248* | 6 | (TG)10 | F  R | TCTGTTTGTTTGGGTCAGGTCCTTC  CGAATACGTATGGAGAACTGCGCATC | Allouis *et al*.,2001 |
| *Xpsmp2249* | 3 | (GT)7imperfect | F  R | CAGTCTCTAACAAACAAACACGGC  GACAGCAACCAACTCCAAACTCCA | Allouis *et al*.,2001 |
| *Xpsmp2251* | 3 | (TG)6 | F  R | TCAAACATAGATATGCCGTGCCTCC  CAGCAAGTCGTGAGGTTCGGATA | Allouis *et al*.,2001 |
| *Xpsmp2267* | 3 | (GA)16 | F  R | GGAAGGCGTAGGGATCAATCTCAC  ATCCACCCGACGAAGGAAACGA | Allouis *et al*.,2001 |
| *Xpsmp2273* | 1 | (GA)12 | F  R | AACCCCACCAGTAAGTTGTGCTGC  GATGACGACAAGACCTTCTCTCC | Allouis *et al*.,2001 |
