# Supplementary material for: Formation of heterotic pools and understanding relationship between molecular divergence and heterosis in pearl millet [Pennisetum glaucum (L.) R. Br.]
Source: PLoS One. 2019 May 7;14(5):e0207463. doi: 10.1371/journal.pone.0207463 (PMC6504090; doi:10.1371/journal.pone.0207463)
Supplement: S3 Table — (DOCX) [file pone.0207463.s003.docx]

**S3 Table. Distribution of 147 pearl millet hybrid parents in different groups based on SSR genotyping, size of groups and number and name of identified representative parents.**

| SSR based cluster | Total number of lines in group | Genetic distance | | Group category | Total number of lines selected | Number of lines selected from | | Representative hybrid parents |
| --- | --- | --- | --- | --- | --- | --- | --- | --- |
|  |  | Min | Max |  |  | B-lines | R-lines |  |
| G1 | 12 | 0.28 | 0.63 | S | 2 | 2 | 0 | B49, B61 |
| G2 | 13 | 0.19 | 0.68 | S | 2 | 2 | 0 | B43, B51 |
| G3 | 35 | 0.18 | 0.72 | L | 3 | 3 | 0 | B18, B23, B26 |
| G4 | 14 | 0.32 | 0.78 | S | 2 | 2 | 0 | B04, B08 |
| G5 | 22 | 0.28 | 0.73 | S | 2 | 0 | 2 | R46, R51 |
| G6 | 16 | 0.27 | 0.63 | S | 2 | 0 | 2 | R44, R66 |
| G7 | 15 | 0.43 | 0.80 | S | 2 | 0 | 2 | R26, R70 |
| G8 | 20 | 0.33 | 0.81 | S | 2 | 0 | 2 | R02, R20 |
