# Supplementary material for: Formation of heterotic pools and understanding relationship between molecular divergence and heterosis in pearl millet [Pennisetum glaucum (L.) R. Br.]
Source: PLoS One. 2019 May 7;14(5):e0207463. doi: 10.1371/journal.pone.0207463 (PMC6504090; doi:10.1371/journal.pone.0207463)
Supplement: S4 Table — (DOCX) [file pone.0207463.s004.docx]

**S4 Table. GCA and SCA of the pearl millet parental lines and hybrids with grain yield of representative hybrid parental lines involved in this study.**

| **Lines** | **B49** | **B26** | **B04** | **B61** | **B23** | **B08** | **B51** | **B43** | **B18** | **R66** | **R02** | **R20** | **R44** | **R26** | **R46** | **R51** | **R70** |
| --- | --- | --- | --- | --- | --- | --- | --- | --- | --- | --- | --- | --- | --- | --- | --- | --- | --- |
| **B49** |  | -792.3** | -279.5 | -602.7** | 154.9 | -25.4 | -54.5 | 509.8** | -82.1 | 663.7** | 455.3* | 301.8 | 942.4** | 804.1** | 521.1** | 678.6** | 1091.0** |
| **B26** |  |  | -272.8 | -674.0** | -1315.6** | 52.0 | 868.1** | 724.8** | -1093.5** | 1522.9** | 440.9* | 570.6** | -10.6 | 786.9** | 656.2** | 391.5* | 428.5* |
| **B04** |  |  |  | 125.9 | 174.0 | 209.1 | 830.3** | -3.1 | -374.4* | 505.6** | 18.3 | 39.4 | 678.3** | 814.2** | -207.4 | -285.3 | 652.6** |
| **B61** |  |  |  |  | -492.7** | 358.4* | -234.3 | 526.5** | -290.3 | -215.3 | 194.5 | 764.2** | 694.7** | 507.3** | 611.5** | 300.9 | 757.7** |
| **B23** |  |  |  |  |  | 173.0 | 415.6* | 186.0 | -913.8** | 1249.6** | 326.9 | 606.1** | 773.9** | 293.2 | 402.9* | 77.7 | 597.2** |
| **B08** |  |  |  |  |  |  | 567.6** | 237.8 | -437.0* | 188.9 | 430.1* | 297.2 | 579.0** | 514.9** | -359.6 | 79.3 | -736.4** |
| **B51** |  |  |  |  |  |  |  | -84.6 | 880.0** | 480.4** | -204.0 | -143.5 | 682.6** | 223.1 | 242.0 | 285.0 | 294.4 |
| **B43** |  |  |  |  |  |  |  |  | 511.5** | -414.2* | 565.5** | 342.2 | -312.3 | 378.7* | 60.2 | -216.3 | 156.2 |
| **B18** |  |  |  |  |  |  |  |  |  | 481.4** | 325.1 | 818.2** | 560.4** | 302.6 | 978.5** | 598.3** | 645.5** |
| **R66** |  |  |  |  |  |  |  |  |  |  | 62.6 | -322.8 | -109.5 | 100.1 | -427.1* | -117.8 | 319.1 |
| **R02** |  |  |  |  |  |  |  |  |  |  |  | -549.6** | 132.9 | -319.9 | 141.4 | -142.9 | 1031.1** |
| **R20** |  |  |  |  |  |  |  |  |  |  |  |  | 24.3 | -371.9* | 128.3 | -38.6 | -380.6* |
| **R44** |  |  |  |  |  |  |  |  |  |  |  |  |  | -505.7** | -472.7** | 71.5 | 391.3* |
| **R26** |  |  |  |  |  |  |  |  |  |  |  |  |  |  | -28.7 | -344.3 | 745.2** |
| **R46** |  |  |  |  |  |  |  |  |  |  |  |  |  |  |  | 529.4** | -275.2 |
| **R51** |  |  |  |  |  |  |  |  |  |  |  |  |  |  |  |  | 223.6 |
| **R70** |  |  |  |  |  |  |  |  |  |  |  |  |  |  |  |  |  |
| **GCA** | 319.6** | 119.0** | -547.0** | -486.4** | -105.1* | -143.0** | 659.6** | -44.2 | -95.7* | 513.0** | 215.6** | -82.6 | 38.4 | -102.0* | -498.0** | -236.5** | 475.2** |
| **Grain yield kg ha^-1^** | 2275.6 | 2874.4 | 1376.4 | 1645.4 | 2226.4 | 2442.1 | 2586.0 | 2118.2 | 2136.4 | 2821.8 | 2756.7 | 2575.3 | 1804.1 | 1633.6 | 1531.7 | 2269.1 | 1767.4 |

*, ** Significant at 0.05, 0.01 levels of probability, respectively
