# Supplementary material for: Formation of heterotic pools and understanding relationship between molecular divergence and heterosis in pearl millet [Pennisetum glaucum (L.) R. Br.]
Source: PLoS One. 2019 May 7;14(5):e0207463. doi: 10.1371/journal.pone.0207463 (PMC6504090; doi:10.1371/journal.pone.0207463)
Supplement: S5 Table — (DOCX) [file pone.0207463.s005.docx]

**S5 Table. Analysis of variance for combining ability.**

| Source of variation | Degree of freedom | Variance Component | Standard error | Z-Value | P-Value |
| --- | --- | --- | --- | --- | --- |
| General Combining ability (GCA) | 16 | 282.69 | 109.03 | 2.59 | 0.0048 |
| Specific Combining ability (SCA) | 135 | 12.4759 | 3.3976 | 3.67 | 0.0001 |
| GCA × Environment | 16 | 3.8306 | 1.9709 | 1.94 | 0.026 |
| SCA × Environment | 135 | 16.7755 | 3.0734 | 5.46 | <.0001 |
