# Supplementary material for: Formation of heterotic pools and understanding relationship between molecular divergence and heterosis in pearl millet [Pennisetum glaucum (L.) R. Br.]
Source: PLoS One. 2019 May 7;14(5):e0207463. doi: 10.1371/journal.pone.0207463 (PMC6504090; doi:10.1371/journal.pone.0207463)
Supplement: S6 Table — (DOCX) [file pone.0207463.s006.docx]

**S6 Table. Better-parent heterosis (above diagonal) and mid-parent heterosis (below diagonal) of pearl millet hybrids.**

| **Lines** | **B49** | **B26** | **B04** | **B61** | **B23** | **B08** | **B51** | **B43** | **B18** | **R66** | **R02** | **R20** | **R44** | **R26** | **R46** | **R51** | **R70** |
| --- | --- | --- | --- | --- | --- | --- | --- | --- | --- | --- | --- | --- | --- | --- | --- | --- | --- |
| **B49** |  | 18.53 | 43.79* | 34.34 | 81.47** | 60.52** | 82.71** | 101.52** | 72.29** | 85.59** | 73.87** | 67.22** | 124.06** | 111.54** | 81.43** | 99.19** | 148.74** |
| **B26** | 32.31* |  | 7.6 | -3.26 | -13.07 | 33.13 | 87.90** | 59.50** | -4.92 | 106.07** | 58.51** | 52.94** | 36.11* | 59.36** | 41.70* | 40.33* | 69.11** |
| **B04** | 79.19** | 45.52** |  | 74.56* | 49.96* | 35.62 | 82.77** | 50.59* | 28.79 | 52.71** | 26.1 | 25.01 | 119.26** | 138.99** | 64.20* | 19.85 | 145.24** |
| **B61** | 55.93** | 23.04 | 90.11** |  | 21.59 | 43.72* | 44.50* | 78.53** | 36.9 | 26.11 | 34.91* | 54.95** | 123.33** | 124.41** | 105.73** | 49.99* | 156.02** |
| **B23** | 83.46** | -2.03 | 85.34** | 39.83* |  | 51.60* | 83.16** | 71.66** | 18.11 | 92.74** | 53.79** | 62.74** | 102.20** | 76.30** | 61.36** | 55.63** | 112.19** |
| **B08** | 66.18** | 43.95** | 73.47** | 71.73** | 58.60** |  | 86.59** | 56.70** | 26.16 | 55.72** | 55.64** | 49.04** | 75.15** | 67.27** | 14.54 | 41.80* | 39.95* |
| **B51** | 94.37** | 97.82** | 138.57** | 76.62** | 96.84** | 91.92** |  | 68.45** | 101.45** | 92.25** | 60.79** | 64.38** | 99.87** | 76.58** | 63.27** | 72.78** | 100.89** |
| **B43** | 108.74** | 83.66** | 82.55** | 100.95** | 75.93** | 67.83** | 85.20** |  | 93.57** | 36.12* | 62.59** | 54.89** | 64.14** | 90.41** | 55.83* | 45.43* | 107.22** |
| **B18** | 77.72** | 9.08 | 56.66** | 54.67** | 20.55 | 34.58* | 120.63** | 94.39** |  | 65.66** | 52.20** | 71.17** | 99.24** | 81.54** | 94.94** | 77.89** | 125.96** |
| **R66** | 105.47** | 107.97** | 105.28** | 59.32** | 115.47** | 66.95** | 100.64** | 55.51** | 88.56** |  | 62.18** | 37.09* | 50.83** | 53.34** | 19.33 | 39.71* | 78.83** |
| **R02** | 90.49** | 61.82** | 68.22** | 68.96** | 70.16** | 65.06** | 65.93** | 83.88** | 71.49** | 64.07** |  | 21.38 | 52.24** | 30.57 | 34.34 | 31.31 | 100.08** |
| **R20** | 77.55** | 61.34** | 62.94** | 89.09** | 74.56** | 53.00** | 64.72** | 69.98** | 87.12** | 43.36** | 25.51* |  | 47.41* | 25.19 | 29.48 | 33.66 | 48.02* |
| **R44** | 149.96** | 67.25** | 148.75** | 133.60** | 123.39** | 101.47** | 135.47** | 77.29** | 116.04** | 84.01** | 84.04** | 73.37** |  | 80.19** | 58.83* | 61.35** | 159.36** |
| **R26** | 146.28** | 103.22** | 159.41** | 125.22** | 103.37** | 100.45** | 116.43** | 115.01** | 105.75** | 94.24** | 63.97** | 53.20** | 89.12** |  | 92.60** | 34.98 | 175.89** |
| **R46** | 116.88** | 84.88** | 72.97** | 113.10** | 91.19** | 40.78* | 105.07** | 80.87** | 127.07** | 54.69** | 72.71** | 62.39** | 71.79** | 98.81** |  | 56.95** | 97.45** |
| **R51** | 99.48** | 56.84** | 49.20** | 73.88** | 57.11** | 47.01** | 84.05** | 50.42** | 83.25** | 54.88** | 44.05** | 42.10** | 79.77** | 56.96** | 87.40** |  | 88.66** |
| **R70** | 180.01** | 109.44** | 175.75** | 165.17** | 136.57** | 62.39** | 138.67** | 125.93** | 147.32** | 119.91** | 143.83** | 75.55** | 162.02** | 186.75** | 111.56** | 112.11** |  |

*, ** Significant at 0.05, 0.01 levels of probability, respectively
